# Impact of pulmonary hypertension on outcomes after TEER in patients suffering from mitral regurgitation

**DOI:** 10.1007/s00392-024-02442-1

**Published:** 2024-04-02

**Authors:** Philippa Jaeger, Ioannis Toskas, Jessica-Kristin Henes, Serhii Shcherbyna, Frederic Schwarz, Miriam Euper, Peter Seizer, Harald Langer, Andreas E. May, Tobias Geisler, Meinrad Gawaz, Jürgen Schreieck, Dominik Rath

**Affiliations:** 1 Department of Cardiology and Angiology, University Hospital Tübingen, Tübingen, Germany; 2 Departmen of Cardiology and Angiology, Ostalb-Klinikum, Aalen, Germany; 3 Department of Medicine, Cardiology, Angiology, Hemostasis and Intensive Care Medicine, University Medical Center Mannheim, Mannheim, Germany; 4 Medizinische Klinik I, Klinikum Memmingen, Mannheim, Germany

**Keywords:** Pulmonary Arterial Hypertension, Outcome Assessment, Mitral valve transcatheter edge-to-edge repair

## Abstract

**Aim:**

Data on associations of invasively determined hemodynamic parameters with procedural success and outcomes in patients suffering from mitral regurgitation (MR) undergoing transcatheter edge-to-edge repair of the mitral valve (M-TEER) is limited.

**Methods and results:**

We enrolled 239 patients with symptomatic MR of grade 2 + , who received M-TEER. All patients underwent extensive pre-interventional invasive hemodynamic measurements via right heart catheterization (mean pulmonary arterial pressure (mPAP), systolic- (PAPsys) and diastolic pulmonary arterial pressure (PAPdia), pulmonary arterial wedge pressure (PAWP), a-wave, v-wave, pulmonary vascular resistance (PVR), transpulmonary pressure gradient (TPG), cardiac index (CI), stroke volume index (SVI)). mPAP and PAWP at baseline were neither associated with procedural success, immediate reduction of MR, nor residual MR after 6 months of follow-up. The composite outcome (All-cause mortality (ACM) and/or heart failure induced rehospitalization (HFH)) and HFH differed significantly after M-TEER when stratified according to mPAP, PAWP, PAPdia, a-wave and v-wave. ACM was not associated with the afore mentioned parameters. Neither PVR, TPG, CI nor SVI were associated with the composite outcome and HFH, respectively. In multivariable analyses, PAWP was independently associated with the composite outcome and HFH. PVR and SVI were not associated with outcomes.

**Conclusion:**

PAWP at baseline was significantly and independently associated with HFH and might serve as a valuable parameter for identifying patients at high risk for HFH after M-TEER. ACM and procedural success were not affected by pulmonary arterial pressure before M-TEER. We suggest that the post-capillary component of PH serves as the driving force behind the risk of HFH.

**Supplementary Information:**

The online version contains supplementary material available at 10.1007/s00392-024-02442-1.

## Introduction

Transcatheter edge-to-edge repair of the mitral valve (M-TEER) has emerged as a safe and less invasive therapeutic alternative in patients with severe mitral regurgitation and at high-risk for surgical mitral valve repair [1–4]. M-TEER is an effective intervention for both primary and secondary mitral valve regurgitation (MR) [1, 4].

Pulmonary hypertension (PH), atrial fibrillation and left ventricular dysfunction are strong predictors of perioperative risk and presence of these co-morbidities worsen prognosis [5].

MR is known to cause PH by chronically increasing the mean left atrial pressure and thereby increasing the filling pressures in the pulmonary circulation. Chronic pulmonary venous congestion leads to fibrotic remodeling of the vessels via vasoconstriction and vascular remodeling, resulting in a further increase in resistance and pressure. Subsequent right ventricular dilation and dysfunction leads to tricuspid valve regurgitation. The resulting postcapillary PH is the most common form of PH [6].

Post-capillary PH, which can be either isolated (IpcPH) or combined with a significant pre-capillary component (CpcPH), is defined by a mPAP > 20 mmHg and a PAWP > 15 mmHg. Pulmonary vascular resistance (PVR) is used to distinguish between IpcPH (PVR ≤ 2 Wood units (WU)) and CpcPH (PVR > 2 WU). Pre-capillary PH is defined by mPAP > 20 mmHg, PAWP ≤ 15 mmHg and PVR > 2 WU [6].

Higher pulmonary arterial pressure (PAP) at baseline is associated with higher long-term mortality when compared to lower PAP in patients undergoing M-TEER [7–10]. However, most studies are based on systolic pulmonary artery pressure (sPAP) obtained by echocardiography [8–10]. Data on invasive hemodynamic measurements in patients who suffered from MR and underwent M-TEER is limited, especially regarding the differentiation of pulmonary hypertension into pre-capillary PH, IpcPH and CpcPH, respectively. Understanding the influence of PH on the outcomes of M-TEER procedures is crucial for optimizing patient selection, procedural planning, and post-procedural care.

Here, we aimed to evaluate associations of invasively determined PAP and its components (mean pulmonary arterial pressure (mPAP), systolic pulmonary arterial pressure (PAPsys), diastolic pulmonary arterial pressure (PAPdia), pulmonary arterial wedge pressure (PAWP), a-wave, v-wave, pulmonary vascular resistance (PVR), transpulmonary pressure gradient (TPG), cardiac index (CI) and stroke volume index (SVI)) with procedural success and clinical outcomes in patients undergoing M-TEER.

## Methods

### Patient cohort

This is a retrospective monocenter study. We consecutively enrolled 239 patients with symptomatic, higher grade mitral valve regurgitation (MR) that were admitted to the Department of Cardiology and Angiology of the University Hospital of Tübingen, Germany, for M-TEER between January 2010 and February 2016 [11]. All echocardiographic parameters in this study were originally assessed in the echocardiographic laboratory of the University Hospital of Tübingen [12–14]. Patients suffered from ischemic or nonischemic heart failure with a left ventricular ejection fraction (LVEF, %) from 15 to 60%. Patients had grade 2 + to grade 4 [1, 8, 15] primary and/or secondary MR and remained symptomatic (New York Heart Association [NYHA] functional class II, III, or IV) despite treatment with stable maximal doses of guideline-directed medical therapy and cardiac resynchronization therapy (if appropriate). All patients underwent right heart catheterization prior to M-TEER. Patients were assessed by a heart team that consisted of a heart-failure specialist, an interventional cardiologist, a cardiothoracic surgeon with expertise in mitral-valve disease and an anesthesiologist with experience in heart failure and cardiac surgery. [16] All patients were treated with the MitraClip® device (Abbott, North Chicago, Illinois, USA). mPAP, PAPsys, PAPdia, PAWP, a-wave, v-wave, PVR, TPG, CI and SVI [17] were determined via right heart catheterization prior to M-TEER [18]. PAWP was measured including v-wave and assessed end-expiratory. We sub-categorized pulmonary hypertension according to the “ESC/ERS Guidelines for the diagnosis and treatment of pulmonary hypertension” as mentioned previously. When PVR (cut-off > 2 WU for pre-capillary PH and CpcPH) [6] was not available, we applied the diastolic pressure gradient (DPG) as well as the transpulmonary pressure gradient (TPG) to differentiate between isolated post-capillary PH (DPG < 7 mmHg, TPG ≤ 12 mmHg) and combined post- and pre-capillary PH (DPG ≥ 7 mmHg, TPG > 12 mmHg) [19, 20]. Most patients gave written informed consent, and for those where it could not be obtained due to logistic issues, the institutional ethics committee approved retrospective data analysis. The study was approved by the ethics committee of the University of Tübingen (270/2011BO1, 237/2018BO2 and 187/2023BO2 respectively). The study complies with the declaration of Helsinki and the good clinical practice guidelines.

### Right heart catheterization

Right heart catheterization was performed via femoral access. A sheath was placed into the femoral vein using Seldinger technique. Then, a Swan-Ganz catheter was passed into the right atrium, the right ventricle and the pulmonary artery using standard manipulations under fluoroscopic control. PAP was recorded, and the catheter was advanced until it plugged a branch of one of the pulmonary arteries and the waveform changed to a PAWP. The catheter was then withdrawn and pressures in the pulmonary arteries, the right ventricle and the right atrium were measured sequentially in resting expiratory position.

### Survival outcomes and prognostic associations

All patients were followed up for 360 days for a primary composite clinical outcome consisting of all-cause mortality (ACM) and/or HFH. Secondary outcomes consisted of the single events ACM or HFH. 25 patients (10.5%) were lost to clinical follow-up. Follow-up echocardiography was performed in 205 patients (85.8%). Only patients with clinical follow-up were included into outcome analyses. We additionally analyzed a best-case (all patients lost to follow-up survived without events) and a worst-case scenario (all patients lost to follow-up suffered from hospitalization due to heart failure and/or deceased). Observed statistical significances between investigated groups did not change substantially when reanalyzed using these approaches.

### Statistical analyses

All statistical analyses were performed with SPSS version 27.0 (IBM, USA) and GraphPad Prism software (GraphPad Software, Inc. USA) as previously described [21]. Non-normally distributed data are presented as median with interquartile range (IQR) or count and percentage as appropriate. Kruskal–Wallis-tests (H-tests) were applied as appropriate to analyze differences between more than two groups. Cox proportional hazard (PH) regression analyses with forward variable selection were applied to investigate associations between survival outcomes and hemodynamic parameters, using clinical factors as covariables. The time-dependent covariate method was used to check the proportional hazard assumption of the model. Survival functions were estimated by Kaplan–Meier curves. The log-rank test was applied to compare survival functions. All statistical tests were two-tailed and statistical significance level was defined as p < 0.05.

## Results

### Baseline characteristics

**Fig. 1:**
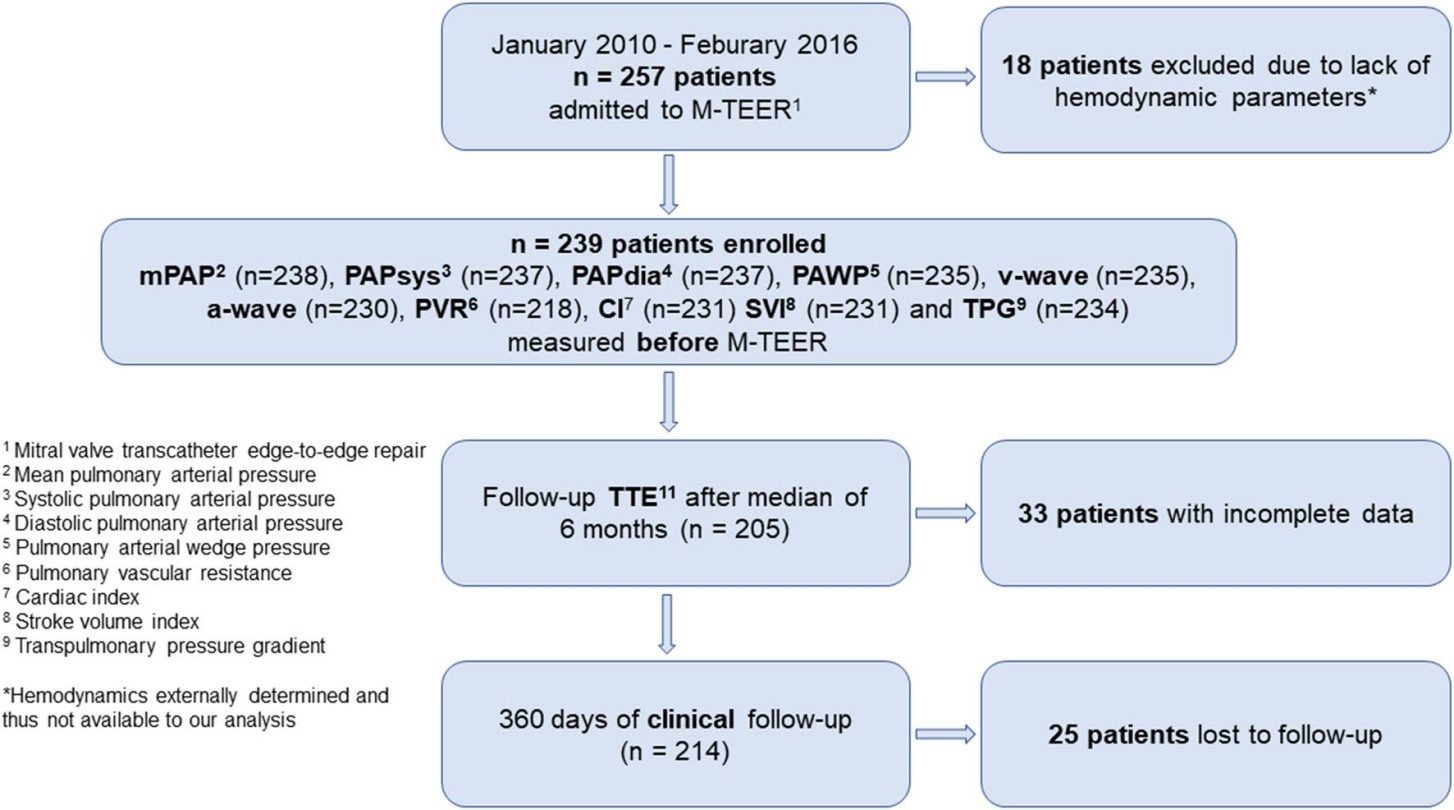
Flowchart of the study cohort

The study flow chart is presented in Fig. 1. Baseline characteristics of the complete clinical cohort stratified according to mPAP quartiles are presented in Table 1. We enrolled 239 patients affected by primary, secondary or combined MR. Of note, in one patient, only PAWP was available. Therefore, Table 1 shows 238 patients. The median age was 78 years, 37.8% were women, 55.5% had degenerative MR, and 85.7% had 3 + MR. Patients with higher mPAP were younger and suffered more often from mild aortic stenosis as well as cardiomyopathies and were more likely to have cardiac resynchronization therapy (CRT). Creatinine levels were higher; however, renal replacement therapy was evenly distributed. Prevalence of concomitant TR, which is related to mPAP and PVR, did not differ significantly between patient groups.

**Table 1:** Baseline clinical, echocardiographic and procedural characteristics of the study population according to baseline mean pulmonary artery pressure

Mean pulmonary arterial pressure						
	Overall (n = 238)	1st quartile (n = 53)	2nd quartile (n = 60)	3rd quartile (n = 64)	4th quartile (n = 61)	p value^a^
Age, median (IQR), y	78 (72–82)	79 (74–85)	78 (74–82)	78 (71–82)	76 (70.0–81.0)	**0.048**
Female, No. (%)	90 (37.8)	23 (43.4)	24 (40.0)	22 (34.4)	21 (34.4)	0.695
BMI, median (IQR), kg	25.0 (22.7–27.9)	25.0 (21.2–28.9)	24.4 (22.3–27.6)	24.6 (22.8–27.7)	26.0 (23.0–28.2)	0.259
Chronic kidney disease, No. (%)	131 (55.0)	25 (47.2)	33 (55.5)	35 (54.7)	38 (62.3)	0.453
Renal replacement therapy, No. (%)	11 (4.6)	1 (1.9)	3 (5.0)	2 (3.1)	5 (8.2)	0.390
NYHA class, No. (%)						
II	33 (13.9)	11 (20.8)	7 (11.7)	5 (7.8)	10 (16.4)	0.200
III	156 (65.5)	31 (58.5)	41 (68.3)	47 (73.4)	37 (60.7)	0.472
IV	49 (20.6)	11 (20.8)	12 (20.0)	12 (18.8)	14 (23.0)	0.950
CAD, No. (%)	177 (74.4)	35 (66.0)	44 (73.3)	54 (84.4)	44 (72.1)	0.140
Cardiomyopathy, No. (%)	125 (52.5)	22 (41.5)	27 (45.0)	37 (57.8)	39 (63.9)	**0.049**
Ischemic cardiomyopathy, No. (%)	84 (35.3)	17 (32.1)	18 (30.0)	24 (37.5)	25 (41.0)	0.577
Atrial fibrillation, No. (%)	167 (70.2)	38 (71.7)	39 (65.0)	45 (70.3)	45 (73.8)	0.684
Atrial flutter, No. (%)	12 (5.0)	4 (7.5)	2 (3.3)	4 (6.3)	2 (3.3)	0.648
Prior mitral valve repair, No. (%)	5 (2.1)	2 (3.8)	1 (1.7)	1 (1.6)	1 (1.6)	0.818
Prior aortic valve repair, No. (%)	27 (11.3)	5 (9.4)	7 (11.7)	9 (14.1)	6 (9.8)	0.848
Prior TAVR, No. (%)	12 (5.0)	1 (1.9)	5 (8.3)	4 (6.3)	2 (3.3)	0.384
Prior CABG, No. (%)	52 (21.8)	10 (18.9)	12 (20.0)	13 (20.3)	11 (27.9)	0.633
Previous cardiogenic shock, No. (%)	15 (6.3)	3 (5.7)	3 (5.0)	5 (7.8)	4 (6.6)	0.927
Permanent pacemaker, No. (%)	16 (6.7)	4 (7.5)	6 (10.0)	4 (6.3)	2 (3.3)	0.520
CRT, No. (%)	36 (15.1)	3 (5.7)	9 (15.0)	9 (14.1)	15 (24.6)	**0.046**
COPD, No. (%)	26 (10.9)	5 (9.4)	3 (5.0)	10 (15.6)	8 (13.1)	0.257
CVRF	Overall (n = 238)	1st quartile (n = 53)	2nd quartile (n = 60)	3rd quartile (n = 64)	4th quartile (n = 61)	p value^a^
Diabetes, No. (%)	71 (29.8)	14 (26.4)	17 (28.3)	16 (25.0)	24 (39.3)	0.294
Smoking, No. (%)	39 (16.4)	7 (13.2)	7 (11.7)	9 (14.1)	16 (26.2)	0.115
Hyperlipidaemia, No. (%)	126 (52.9)	23 (43.4)	34 (56.7)	35 (54.7)	34 (55.7)	0.468
Hypertension, No. (%)	190 (79.8)	41 (77.4)	46 (76.7)	49 (76.6)	54 (88.5)	0.277
Medication at admission	Overall (n = 238)	1st quartile (n = 53)	2nd quartile (n = 60)	3rd quartile (n = 64)	4th quartile (n = 61)	p value^a^
Betablocker, No. (%)	206 (86.6)	46 (86.8)	23 (88.3)	52 (81.3)	55 (30.2)	0.494
Digitoxin, No. (%)	26 (10.9)	5 (9.4)	8 (13.3)	7 (10.9)	6 (9.8)	0.907
ACE-I, No. (%)	152 (63.9)	34 (64.2)	41 (68.3)	36 (56.3)	41 (67.2)	0.489
ARBs, No. (%)	43 (18.1)	9 (17.0)	8 (13.3)	11 (17.2)	15 (24.6)	0.434
Spironolactone, No. (%)	58 (24.4)	10 (18.9)	14 (23.3)	18 (28.1)	16 (26.2)	0.680
Eplerenone, No. (%)	71 (29.8)	14 (26.4)	21 (35.0)	14 (21.9)	22 (36.1)	0.248
Torasemid, No. (%)	195 (81.9)	45 (84.9)	52 (86.7)	47 (73.4)	51 (83.6)	0.216
HCT, No. (%)	14 (5.9)	5 (9.4)	3 (5.0)	3 (4.7)	3 (4.9)	0.669
Xipamide, No. (%)	38 (16.0)	5 (9.4)	9 (15.0)	10 (15.6)	14 (23.0)	0.267
Laboratory parameters at admission, median (IQR)	Overall (n = 238)	1st quartile (n = 53)	2nd quartile (n = 60)	3rd quartile (n = 64)	4th quartile (n = 61)	p value^a^
Hb (g/dl)	12.1 (10.8–13.2)	12.1 (11.0–13.2)	12.3 (11.0–13.3)	11.3 (10.1–13.1)	12.3 (11.1–13.5)	0.069
eGFR (ml/min/1,73m2)	52.6 (38.9–64.2)	52.0 (39.3–70.3)	54.1 (46.7–67.4)	48.1 (37.2–59.7)	51.2 (36.0–59.7)	0.066
Creatinine (mg/dl)	1.2 (1.0–1.7)	1.2 (0.8 -1.5)	1.1 (1.0–1.3)	1.3 (1.0–1.8)	1.3 (1.0–1.8)	**0.033**
CRP (mg/dl)	0.6 (0.1–1.8)	0.4 (0.1–1.8)	0.5 (0.1–1.3)	0.7 (0.2–1.9)	0.7 (0.2–1.8)	0.543
TnI (µg/l)	0.03 (0.03–0.05)	0.03 (0.02–0.04)	0.04 (0.03–0.06)	0.03 (0.03–0.06)	0.04 (0.03–0.05)	0.494
NT-proBNP (ng/l)	5086 (1901–10751)	4161 (1914–13,861)	4883 (1084–8331)	5093 (2269–11,247)	5934 (2851- 10,340)	0.875
Echocardiographic parameters at admission	Overall (n = 238)	1st quartile (n = 53)	2nd quartile (n = 60)	3rd quartile (n = 64)	4th quartile (n = 61)	p value^a^
Mitral regurgitation, No. (%)						
2 + (Moderate to severe)	32 (13.4)	4 (7.5)	12 (20.0)	8 (12.5)	8 (13.1)	0.278
3 + (Severe)	168 (70.6)	43 (81.1)	46 (76.7)	43 (67.2)	36 (59.0)	**0.042**
4 + (Massive)	36 (15.1)	6 (11.3)	2 (3.3)	13 (20.3)	15 (24.6)	**0.005**
Aortic regurgitation grade, No. (%)						
1 + (Mild)	120 (50.4)	26 (49.1)	32 (53.3)	34 (53.1)	28 (45.9)	0.819
2 + (Moderate)	3 (1.3)	0 (0)	1 (1.7)	2 (3.1)	0 (0)	0.345
Aortic stenosis, No. (%)	25 (10.5)	5 (9.4)	5 (8.3)	3 (4.7)	12 (19.7)	**0.025**
1 + (Mild)	16 (6.7)	3 (5.7)	1 (1.7)	3 (4.7)	9 (14.8)	**0.026**
Tricuspid reguritation, No. (%)						
2 + (Moderate)	77 (32.4)	12 (22.6)	19 (31.7)	24 (37.5)	22 (36.1)	0.312
3 + (Severe)	16 (6.7)	2 (3.8)	4 (6.7)	8 (12.5)	2 (3.3)	0.156
LVEF, median (IQR)	38 (30–50)	40 (30–55)	40 (30–50)	41 (30–54)	35 (28–45)	0.058
LVEF, No. (%)						
< 40% (HFrEF)	137 (57.6)	26 (49.1)	34 (56.7)	32 (50.0)	44 (72.1)	0.051
41%-49% (HFmrEF)	22 (9.2)	5 (9.4)	6 (10.0)	6 (9.4)	5 (8.2)	0.989
> 50% (HFpEF)	79 (33.2)	21 (39.6)	20 (33.3)	26 (40.6)	12 (19.7)	0.055
Etiology of mitral regurgitation, No (%)						
Primary only	88 (36.8)	22 (41.5)	25 (41.7)	24 (37.5)	17 (27.9)	0.360
Secondary only	132 (55.5)	28 (52.8)	32 (53.3)	34 (53.1)	38 (62.3)	0.670
Combined	18 (7.5)	3 (5.7)	5 (5.0)	6 (9.4)	6 (9.8)	0.662

^a^Calculated by using the Kruskal–Wallis test for continuous variables and Pearson χ^2^ test for categorical variables

Abbreviations: ACE-I, angiotensin converting-enzyme inhibitors; ARBs, angiotensin receptor blockers; BMI, body mass index; CABG, coronary artery bypass graft; CAD, coronary artery disease; COPD, chronic obstructive pulmonary disease; CRP, C-reactive protein; CRT, cardiac resynchronization therapy; CVRF, cardiovascular risk factors; eGFR, estimated glomerular filtration rate; Hb, hemoglobin; HFmrEF, heart failure with mildly reduced ejection fraction; HFpEF, heart failure with preserved ejection fraction; HFrEF, heart failure with reduced ejection fraction; LVEF, left ventricular ejection fraction; NT-proBNP, N-terminal pro brain natriuretic peptide; NYHA, New York Heart Association; TAVR, transcatheter aortic valve replacement;

TnI, troponin I

### Procedural success

**Fig. 2:**
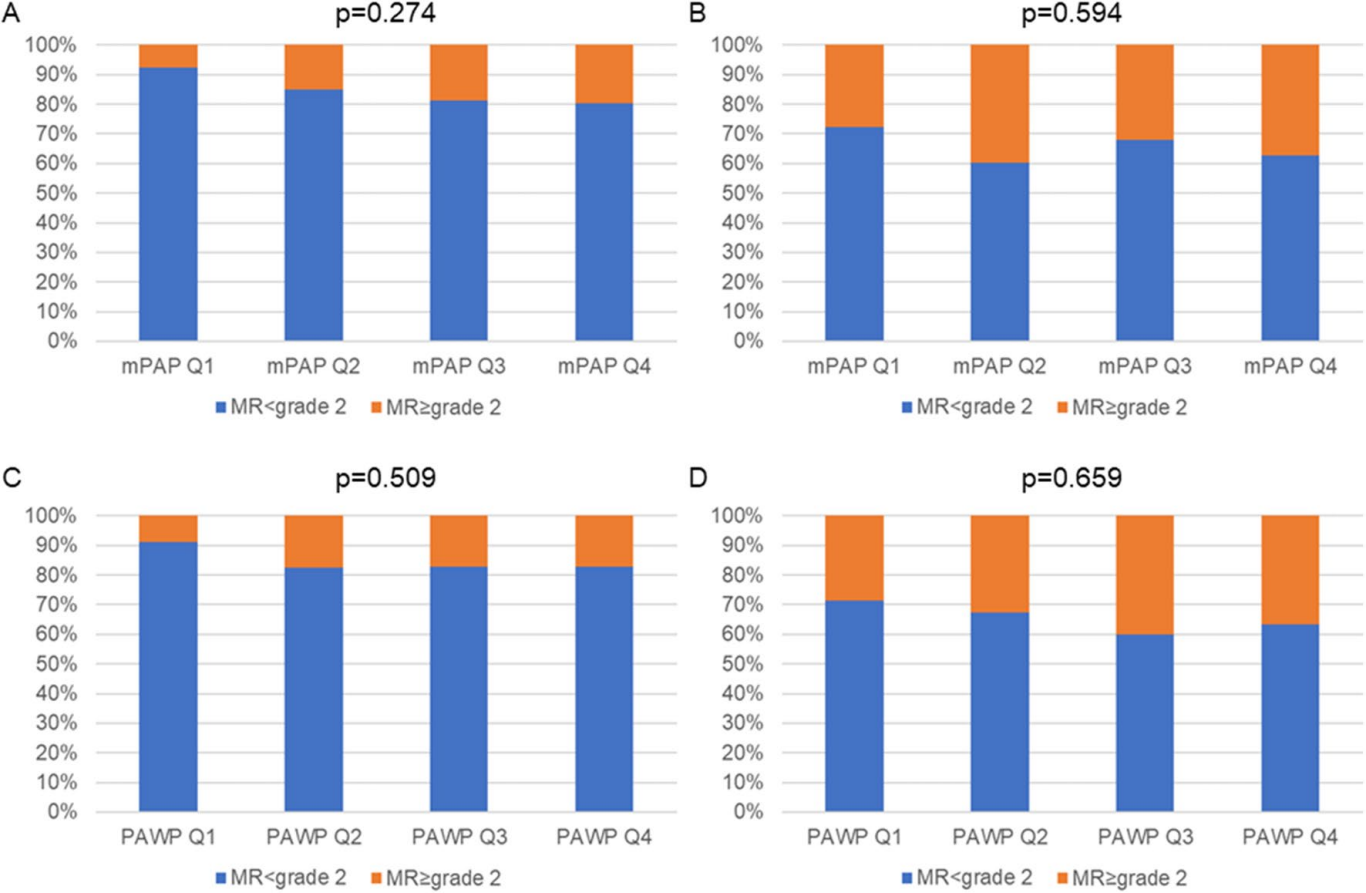
Good procedural result (MR < grade 2) immediately after M-TEER and at 6-months follow-up stratified according to mPAP and PAWP at baseline. A and C: MR < grade 2 (%) immediately after M-TEER. B and D: MR < grade 2 (%) after 6 months of follow-up. mPAP Q1 < 24, mPAP Q2 ≥ 24 < 30, mPAP Q3 ≥ 30 < 37, mPAP Q4 ≥ 37 mmHg. PAWP Q1 < 12, PAWP Q2 ≥ 12 < 18, PAWP Q3 ≥ 18 < 25, PAWP Q4 ≥ 25 mmHg. Abbreviations: mPAP, mean pulmonary arterial pressure; MR, mitral valve regurgitation; M-TEER, transcatheter edge-to-edge repair of the mitral valve; PAWP, pulmonary arterial wedge pressure; Q, quartile.

The MitraClip® procedure was completed in all patients. After M-TEER, MR was reduced to mild or less in 200 patients (83.7%), to moderate in 33 patients (13.8%) while there was no relevant reduction of MR in 6 patients (2.5%). After 6 months of follow-up, 134 patients had MR of mild or less severity (65.4%), 61 patients had moderate MR (29.8%) whereas 10 patients hat severe MR (4.9%). MR grade IV at baseline was associated with higher mPAP (3rd and 4th quartile) before M-TEER. Immediate reduction of MR and success of the procedure (MR < grade 2) were not associated with hemodynamic parameters before M-TEER (Table 2, Fig. 2).

**Table 2:** Good procedural result (MR < grade 2) immediately after M-TEER and at 6-months follow-up stratified according to quartiles (Q) of hemodynamic parameters

MR < grade 2 after M-TEER (%)				
mPAP Q1	mPAP Q2	mPAP Q3	mPAP Q4	p-value
49 (92.5%)	51 (85.0%)	52 (81.3%)	49 (80.3%)	0.274
PAPsys Q1	PAPsys Q2	PAPsys Q3	PAPsys Q4	
51 (92.7%)	48 (88.9%)	51 (75.0%)	50 (84.7%)	0.038
PAPdia Q1	PAPdia Q2	PAPdia Q3	PAPdia Q4	
41 (87.2%)	57 (86.4%)	51 (81.0%)	52 (85.2%)	0.783
PAWP Q1	PAWP Q2	PAWP Q3	PAWP Q4	
51 (91.1%)	47 (82.5%)	53 (82.8%)	48 (82.8%)	0.509
a-wave Q1	a-wave Q2	a-wave Q3	a-wave Q4	
48 (88.9%)	46 (85.2%)	49 (84.5%)	51 (81.0%)	0.699
v-wave Q1	v-wave Q2	v-wave Q3	v-wave Q4	
51 (87.7%)	51 (87.9%)	49 (81.7%)	49 (81.7%)	0.523
PVR Q1	PVR Q2	PVR Q3	PVR Q4	
47 (87.0%)	47 (85.5%)	47 (88.7%)	43 (76.8%)	0.316
TPG Q1	TPG Q2	TPG Q3	TPG Q4	
51 (87.9%)	48 (90.6%)	47 (79.7%)	53 (82.8%)	0.355
CI Q1	CI Q2	CI Q3	CI Q4	
45 (80.4%)	60 (88.2%)	40 (85.1%)	51 (85.0%)	0.685
SVI Q1	SVI Q2	SVI Q3	SVI Q4	
45 (81.8%)	49 (86.0%)	50 (87.7%)	47 (82.5%)	0.794
MR < grade 2 after 6 months (%)				
mPAP Q1	mPAP Q2	mPAP Q3	mPAP Q4	p-value
34 (72.3%)	32 (60.4%)	36 (67.9%)	32 (62.7%)	0.594
PAPsys Q1	PAPsys Q2	PAPsys Q3	PAPsys Q4	
32 (68.1%)	35 (72.9%)	33 (58.9%)	34 (65.4%)	0.499
PAPdia Q1	PAPdia Q2	PAPdia Q3	PAPdia Q4	
26 (63.4%)	42 (70.0%)	34 (63.0%)	32 (65.3%)	0.857
PAWP Q1	PAWP Q2	PAWP Q3	PAWP Q4	
35 (71.4%)	35 (67.3%)	30 (60.0%)	33 (63.5%)	0.659
a-wave Q1	a-wave Q2	a-wave Q3	a-wave Q4	
31 (67.4%)	34 (70.8%)	30 (62.5%)	33 (60.0%)	0.666
v-wave Q1	v-wave Q2	v-wave Q3	v-wave Q4	
34 (68.0%)	33 (67.3%)	31 (62.0%)	36 (66.7%)	0.920
PVR Q1	PVR Q2	PVR Q3	PVR Q4	
33 (70.2%)	31 (66.0%)	34 (66.7%)	27 (58.7%)	0.697
TPG Q1	TPG Q2	TPG Q3	TPG Q4	
30 (58.8%)	32 (66.7%)	36 (70.6%)	35 (67.3%)	0.639
CI Q1	CI Q2	CI Q3	CI Q4	
27 (60.0%)	38 (65.5%)	31 (72.1%)	36 (67.9%)	0.677
SVI Q1	SVI Q2	SVI Q3	SVI Q4	
28 (62.2%)	35 (71.4%)	36 (66.7%)	33 (64.7%)	0.808

CI, cardiac index; mPAP, mean pulmonary arterial pressure; MR, mitral valve regurgitation; M-TEER, transcatheter edge-to-edge repair of the mitral valve; PAPdia, diastolic pulmonary arterial pressure; PAPsys, systolic pulmonary arterial pressure; PAWP, pulmonary arterial wedge pressure; PVR, pulmonary vascular resistance; Q, quartiles; SVI, stroke volume index; TPG, transpulmonary pressure gradient

### Outcomes

**Fig. 3:**
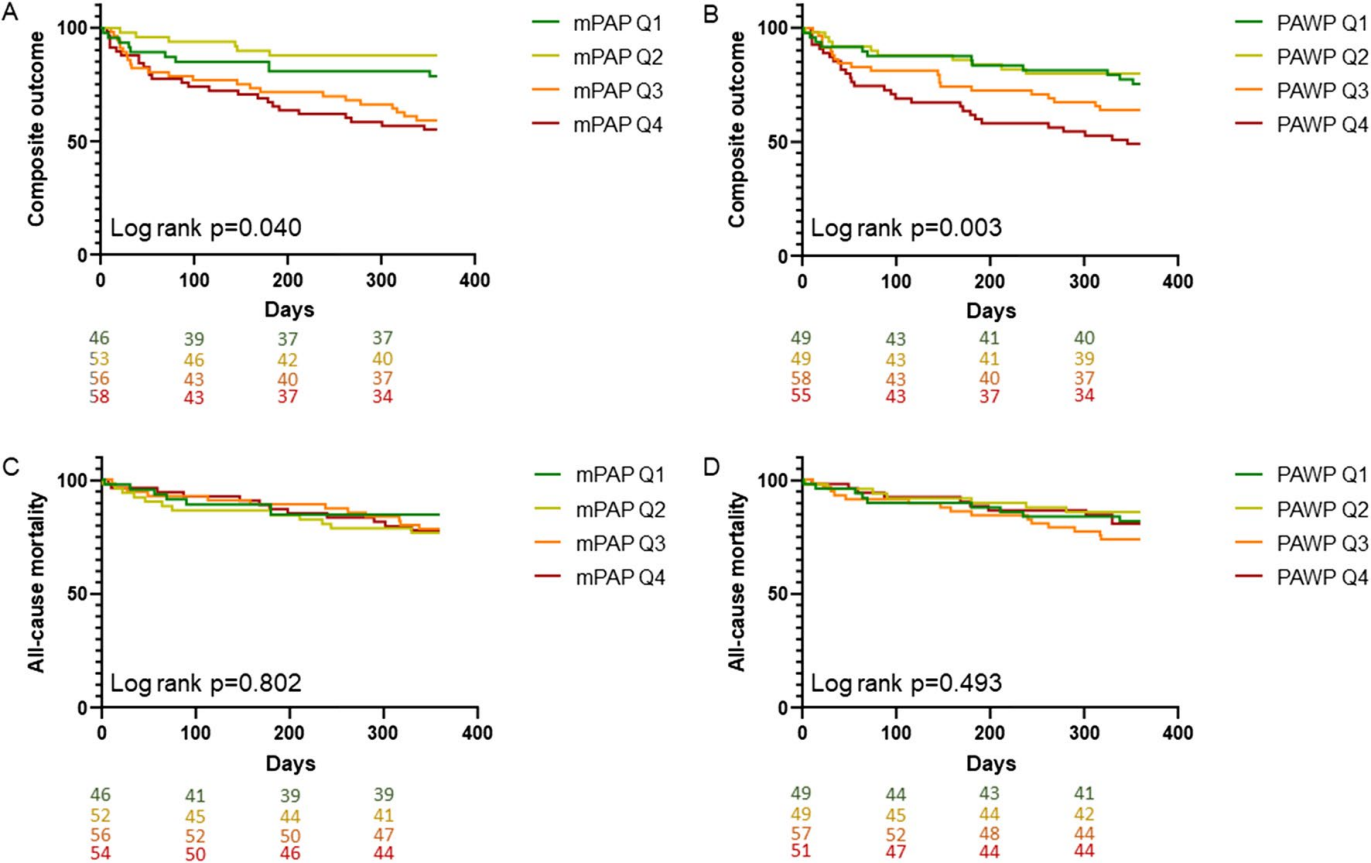
Kaplan–Meier estimates showing composite outcome (A, B) and ACM (C, D) stratified according to mPAP and PAWP at baseline. mPAP Q1 < 24, mPAP Q2 ≥ 24 < 30, mPAP Q3 ≥ 30 < 37, mPAP Q4 ≥ 37 mmHg. PAWP Q1 < 12, PAWP Q2 ≥ 12 < 18, PAWP Q3 ≥ 18 < 25, PAWP Q4 ≥ 25 mmHg. Abbreviations: ACM, all-cause mortality; mPAP, mean pulmonary arterial pressure; PAWP, pulmonary arterial wedge pressure; Q, quartiles.

**Fig. 4:**
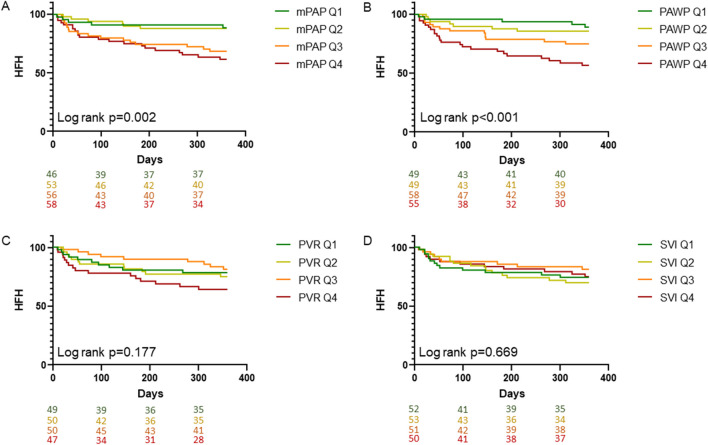
Kaplan–Meier estimates showing HFH stratified according to mPAP (A), PAWP (B), PVR (C) and SVI (D), respectively, at baseline. mPAP Q1 < 24, mPAP Q2 ≥ 24 < 30, mPAP Q3 ≥ 30 < 37, mPAP Q4 ≥ 37 mmHg. PAWP Q1 < 12, PAWP Q2 ≥ 12 < 18, PAWP Q3 ≥ 18 < 25, PAWP Q4 ≥ 25 mmHg. PVR Q1 < 1.8, PVR Q2 ≥ 1.8 < 2.7, PVR Q3 ≥ 2.7 < 4.3, PVR Q4 ≥ 4.3 WU. SVI Q1 < 23.7, SVI Q2 ≥ 23.7 < 31.3, SVI Q3 ≥ 31.3 < 41.8, SVI Q4 ≥ 41.8 ml/m^2^. Abbreviations: HFH, heart failure induced rehospitalization; mPAP, mean pulmonary arterial pressure; PAWP, pulmonary arterial wedge pressure; PVR, pulmonary vascular resistance; Q, quartiles; SVI, stroke volume index

All-cause mortality did not differ significantly after the MitraClip® procedure stratified according to hemodynamic parameters before M-TEER. The composite outcome and HFH were, however, significantly associated with mPAP, PAWP, PAPdia, a-wave and v-wave, respectively. Of note, neither PVR, TPG, CI nor SVI were associated with the composite outcome, ACM and HFH, respectively (Table 3 and Figs. 3 and 4). Range of hemodynamic parameters in quartiles 1–4 is presented in Table 3. While the incidence of HFH was higher in the 3rd and 4th mPAP quartile at baseline when compared to the 1st and 2nd quartile, PAWP showed a linear trend towards higher pressure being associated with HFH. In multivariable analyses, PAWP at baseline remained independently associated with the composite outcome and HFH after adjustment for covariates (Table 4).

**Table 3:** Number of events, patients at risk and incidence rate/100 person years for the composite outcome, ACM and HFH stratified according to quartiles of hemodynamic parameters

Event	mPAP 1st quartile	mPAP 2nd quartile	mPAP 3rd quartile	mPAP 4th quartile	Log rank p
Composite outcome	10/46/21.7	14/53/26.4	23/56/41.1	26/59/44.1	**0.040**
ACM	7/46/15.2	12/52/23.1	12/56/21.4	12/54/22.2	0.802
Hospitalization due to heart failure	5/46/10.9	6/53/11.3	17/56/30.4	21/58/36.2	**0.002**
	PAPsys 1st quartile	PAPsys 2nd quartile	PAPsys 3rd quartile	PAPsys 4th quartile	
Composite outcome	15/48/31.3	9/47/19.1	25/59/42.4	23/57/40.4	0.066
ACM	13/48/27.1	4/46/8.7	14/58/24.1	11/54/20.4	0.261
Hospitalization due to heart failure	6/48/12.5	5/47/10.6	18/59/30.5	20/57/35.1	**0.006**
	PAPdia 1st quartile	PAPdia 2nd quartile	PAPdia 3rd quartile	PAPdia 4th quartile	
Composite outcome	9/39/23.1	14/60/23.3	26/54/48.1	23/59/39.0	**0.028**
ACM	6/39/15.4	9/60/15.0	16/52/30.8	11/56/19.6	0.208
Hospitalization due to heart failure	5/39/12.8	9/60/15.0	18/54/33.3	17/59/28.8	**0.042**
	PAWP 1st quartile	PAWP 2nd quartile	PAWP 3rd quartile	PAWP 4th quartile	
Composite outcome	12/49/23.1	10/49/20.4	21/58/36.2	28/55/50.9	**0.003**
ACM	9/49/15.4	7/49/15.0	15/57/30.8	10/51/19.6	0.493
Hospitalization due to heart failure	5/49/10.2	7/49/14.3	14/58/24.1	23/55/41.8	** < 0.001**
	a-wave 1st quartile	a-wave 2nd quartile	a-wave 3rd quartile	a-wave 4th quartile	
Composite outcome	16/45/35.6	8/48/16.7	15/51/29.4	31/61/50.8	**0.002**
ACM	12/45/26.7	7/48/14.6	9/50/18.0	13/57/22.8	0.452
Hospitalization due to heart failure	9/45/20.0	2/48/4.2	12/51/23.5	25/61/41.0	** < 0.001**
	v-wave 1st quartile	v-wave 2nd quartile	v-wave 3rd quartile	v-wave 4th quartile	
Composite outcome	13/48/27.1	9/50/18.0	20/56/35.7	29/57/50.9	**0.002**
ACM	10/48/20.8	5/50/10.0	13/56/23.2	13/52/25.0	0.285
Hospitalization due to heart failure	5/49/10.4	6/48/12.0	15/50/26.8	23/57/40.4	** < 0.001**
	PVR 1st quartile	PVR 2nd quartile	PVR 3rd quartile	PVR 4th quartile	
Composite outcome	15/49/30.6	16/50/32.0	14/50/28.0	20/47/42.6	0.304
ACM	6/49/12.2	11/48/22.9	9/50/18.0	10/45/22.2	0.531
Hospitalization due to heart failure	10/49/20.4	12/50/24.0	9/50/18.0	16/47/40.4	0.177
	TPG 1st quartile	TPG 2nd quartile	TPG 3rd quartile	TPG 4th quartile	
Composite outcome	23/56/41.1	11/47/23.4	17/51/33.3	20/56/35.7	0.365
ACM	9/54/16.7	9/46/19.6	11/50/22.0	12/55/21.8	0.924
Hospitalization due to heart failure	17/56/30.4	6/47/24.0	11/51/18.0	15/56/40.4	0.177
	CI 1st quartile	CI 2nd quartile	CI 3rd quartile	CI 4th quartile	
Composite outcome	21/50/42.0	21/63/33.3	15/42/35.7	13/51/25.5	0.458
ACM	14/49/28.6	12/59/20.3	8/42/19.0	6/51/11.8	0.261
Hospitalization due to heart failure	17/50/34.0	10/63/15.9	12/42/28.6	10/51/19.6	0.198
	SVI 1st quartile	SVI 2nd quartile	SVI 3rd quartile	SVI 4th quartile	
Composite outcome	19/52/36.0	21/53/38.5	14/51/27.5	16/50/32.0	0.637
ACM	12/50/24.0	12/51/23.5	8/51/15.7	8/49/16.3	0.630
Hospitalization due to heart failure	13/52/25.0	15/53/28.3	9/51/17.6	12/50/24.0	0.669

ACM, all-cause mortality; CI, cardiac index; mPAP, mean pulmonary arterial pressure; PAPdia, diastolic pulmonary arterial pressure; PAPsys, systolic pulmonary arterial pressure; PAWP, pulmonary arterial wedge pressure; PVR, pulmonary vascular resistance; SVI, stroke volume index; TPG, transpulmonary pressure gradient

*mPAP Q1* < 24, *mPAP Q2* ≥ 24 < 30, *mPAP Q3* ≥ 30 < 37, *mPAP Q4* ≥ 37 mmHg

*PAPsys* Q1 < 39, *PAPsys Q2* ≥ 39 < 48, *PAPsys Q3* ≥ 48 < 59.5, *PAPsys Q4* ≥ 59.5 mmHg

*PAPdia Q1* < 11, *PAPdia* Q2 ≥ 11 < 17, *PAPdia* Q3 ≥ 17 < 22, *PAPdia Q4* ≥ 22 mmHg

*PAWP Q1* < 12, *PAWP Q2* ≥ 12 < 18, *PAWP Q3* ≥ 18 < 25, *PAWP Q4* ≥ 25 mmHg

*a-wave Q1* < 15, *a-wave Q2* ≥ 15 < 28, *a-wave Q3* ≥ 22 < 28, *a-wave Q4* ≥ 28 mmHg

*v-wave Q1* < 17, *v-wave Q2* ≥ 17 < 26, *v-wave Q3* ≥ 26 < 36, *v-wave Q4* ≥ 36 mmHg

*PVR Q1* < 1.8, *PVR Q2* ≥ 1.8 < 2.7, *PVR Q3* ≥ 2.7 < 4.3, *PVR Q4* ≥ 4.3 WU

*TPG Q1* < 8.8, *TPG Q2* ≥ 8.8 < 12, *TPG Q3* ≥ 12 < 16, *TPG Q4* ≥ 16 mmHg

*CI Q1* < 1.8, *CI Q2* ≥ 1.8 < 2.2, *CI Q3* ≥ 2.2 < 2.8, *CI Q4* ≥ 2.8 l/min/m^2^

*SVI Q1* < 23.7, *SVI Q2* ≥ 23.7 < 31.3, *SVI Q3* ≥ 31.3 < 41.8, *SVI Q4* ≥ 41.8 ml/m^2^

**Table 4:** Cox regression analyses with forward variable selection showing independent associations of PAWP with the composite outcome and HFH after adjustment for covariates

Variable	HR (95% CI)	p (Composite outcome)
Hb	0.81 (0.71–0.92)	0.001
CRT	1.93 (1.09–3.43)	0.025
PAWP quartiles	1.31 (1.04–1.64)	0.022
Variable	HR (95% CI)	p (HFH)
PAWP quartiles	1.75 (1.31–2.33)	< 0.001

AS, aortic stenosis; CMP, cardiomyopathy; CRT, cardiac resynchronization therapy; Hb, hemoglobin; HFH, heart failure induced rehospitalization; HR, hazard ratio; LVEF, left ventricular ejection fraction; mPAP, mean pulmonary arterial pressure; MR, mitral valve regurgitation; PAPdia, diastolic pulmonary arterial pressure; PAPsys, systolic pulmonary arterial pressure; PAWP, pulmonary arterial wedge pressure; TR, tricuspid regurgitation

Variables included into the model: Age, CMP, CRT, Hb, creatinine, MR, AS, TR, LVEF, mPAP, PAPsys, PAPdia, PAWP, a-wave and v-wave

### Classification of PH

205 patients suffered from pulmonary hypertension (85.8%). Out of these patients, 59 (28.8%) suffered from pre-capillary PH, 53 (25.9%) from IpcPH and 91 (44.4%) from CpcPH. Within these subgroups, 2 patients without PH (6.7%), 8 patients with pre-capillary PH (15.7%), 13 patients with IpcPH (26.5%) and 26 patients with combined CpcPH (31.0%) were hospitalized due to heart failure. Of note, neither PVR nor TPG were associated with outcomes whereas parameters suggestive of left heart disease such as v-wave and PAWP were. Hence, we suggest that the post-capillary component of pulmonary hypertension serves as the driving force behind the risk of recurrent hospitalization due to heart failure.

A limitation of the current study is incomplete data on PVR. Hence, we provide data on patients with PVR available in the supplementary material.

## Discussion

The current study revealed that (1) pulmonary arterial pressure before M-TEER was neither associated with procedural success nor reduction of mitral regurgitation after M-TEER. (2) Pulmonary arterial wedge pressure at baseline was significantly and independently associated with the composite outcome and heart failure induced rehospitalization after M-TEER. (3) Pulmonary arterial pressure was not associated with all-cause mortality.

Previous studies have shown that higher pulmonary arterial pressure is associated with a worse prognosis in patients undergoing M-TEER when compared to those without significant pulmonary hypertension. Tigges et al. [9] evaluated the efficacy of MitraClip® therapy in patients without, with mild and severe pulmonary hypertension, respectively, based on echocardiographically determined systolic pulmonary arterial pressure levels. Similar to our current findings, they showed that interventional success and reduction of MR were achieved in all groups. Our study demonstrated comparable findings based on invasively measured pulmonary arterial pressure. Neither mPAP nor PAWP at baseline were associated with procedural success or with the reduction of MR immediately after M-TEER or at 6 months of follow-up, suggesting M-TEER to be an effective option even for patients with severe PH.

It is well known, that PH is associated to HFH and ACM. In our current study, PH was associated to HFH but not to ACM, which seems to be contradictory. However, we believe, that there are good reasons that may explain our findings. First and foremost, a longer than 360-days follow-up period may have yielded to differences in ACM. This hypothesis is in our opinion supported by other studies in this field. Matsumoto et al. albeit offering a smaller sample size than the current investigation, showed a significant difference in ACM stratified according to PH. However, the Kaplan–Meier estimates in this study showed no difference after 360 days of follow-up but a significant difference after 720 days of follow-up [8]. In a sub-study of the COAPT trial, follow-up for ACM was 24 months. Differences in ACM stratified according to PAPsys were most pronounced after 24 months of follow-up even tough trends were evident after 12 months of follow-up. Interestingly, hospitalization for heart failure within 1-year prior to study inclusion did not differ between patients with PAPsys > 50 mmHg vs PAPsys < 50 mmHg at study inclusion [10]. Tehrani et al. could show an association of an immediate increase of mPAP after M-TEER with HFH but not ACM in a 12 months follow-up. Again, the sample size was small [22]. On the other hand, Tigges et al. found an effect of PAPsys on ACM but not rehospitalization [9]. In a large retrospective analysis by Al-Bawardy and colleagues, associations of elevated pulmonary arterial pressure with both HFH and ACM were clearly demonstrated. There are differences and similarities in these patients when compared to our cohort. In the study by Al-Bawardy, patients tended to be of older age and suffered more frequently from primary MR, respectively. Interestingly, in our cohort, significantly more patients with higher mPAP had undergone cardiac resynchronization therapy (CRT) prior to study inclusion when compared to those with lower mPAP, which may have influenced outcomes. To the best of our knowledge, information on CRT is not available in the study by Al-Bawardy et al.. Of note, over 4000 patients were included into this analysis increasing the statistical power significantly [7].

Several studies show that PH in left heart disease is associated to ACM with patients hospitalized due to HF having mortality rates significantly higher than patients never hospitalized [23, 24]. However, a considerable amount of these studies is dated with newer therapeutic strategies addressing heart failure (e.g. CRT, state of the art medication) not yet available and/or offer follow-up exceeding 12 months by far [25–27]. Cappola et al. e.g. state that “among patients with myocarditis, mPA is particularly good at predicting death at 1 year, whereas its prognostic value is much less among other cardiomyopathies” [26]. If we compare the current study to landmark trials in heart failure like the DAPA-HF Trial [28], we see that effects on HFH especially at 360 days of follow-up are much more pronounced than effects on ACM. Also, here, follow-up was 24 months. In a large international cohort of patients hospitalized for HF, prior HF hospitalization was associated with increased mortality in unadjusted and partially adjusted analyses but was not independently associated with 180-day mortality after adjustment for patient characteristics. In this study, 180-days ACM was 11.9% in patients without prior HFH vs. 15.5% in those with prior HFH, respectively [29]. Hence, we suggest that the combination of a limited follow-up period, a moderate sample size, and state of the art therapy are key factors, why PH and HFH are not associated to ACM in the current collective.

We could show that PAWP at baseline remained independently associated with the composite outcome and recurrent HFH after adjustment for covariates. In our analysis, PAWP at baseline was the strongest predictor of HFH, which we consider novel and a strength of our current investigation.

Most previous studies defined pulmonary hypertension based on systolic PAP assessed in transthoracic echocardiography giving an incomplete evaluation of the hemodynamic situation. Our retrospective study tried to overcome these limitations by only including patients with right heart catheterization prior to M-TEER. Thus, we provide more subtle information on pulmonary hypertension than can be given by echocardiographic measurements which may be biased by image quality or presence and severity of tricuspid regurgitation. Furthermore, we can evaluate the effect of postcapillary pulmonary hypertension on prognosis, which is hardly possible in echocardiographic measurements.

In conclusion, pulmonary arterial wedge pressure at baseline might serve as a valuable parameter for identifying patients at high risk for HFH even after successful M-TEER. Hence, patients with high pulmonary arterial wedge pressure before M-TEER might benefit from intensified monitoring and a more stringent medical therapy after intervention to avoid recurrent hospitalization.

## Limitations

The current study has several limitations. First, this is a retrospective study. Hence, the design is susceptible for bias and misinterpretation. Second, the number of included patients is moderate and the study was conducted at a single center. Third, the study collective was rather heterogenous consisting of patients with ischemic and non-ischemic cardiomyopathy as well as primary and secondary MR or a combination of both. This, however, also reflects a “real-world” setting. Fourth, our study does not include a prospective validation cohort. Fifth, a considerable number of patients was lost to follow-up. Finally, a major limitation of the current study is incomplete data on PVR. However, results did not change substantially if only patients with PVR available were analyzed.

## Supplementary Information

Below is the link to the electronic supplementary material.

Supplementary file1 (TIF 1157 KB)

Supplementary file2 (TIF 755 KB)

Supplementary file3 (DOCX 15 KB)

## Data Availability

The data that support the findings of this study are not openly available due to reasons of sensitivity and are available from the corresponding author upon reasonable request.
