# Supplementary figures and images for: Impact of pulmonary hypertension on outcomes after TEER in patients suffering from mitral regurgitation
Source: Clin Res Cardiol. 2024 Apr 2;114(2):203–14. doi: 10.1007/s00392-024-02442-1 (PMC11839688; doi:10.1007/s00392-024-02442-1)

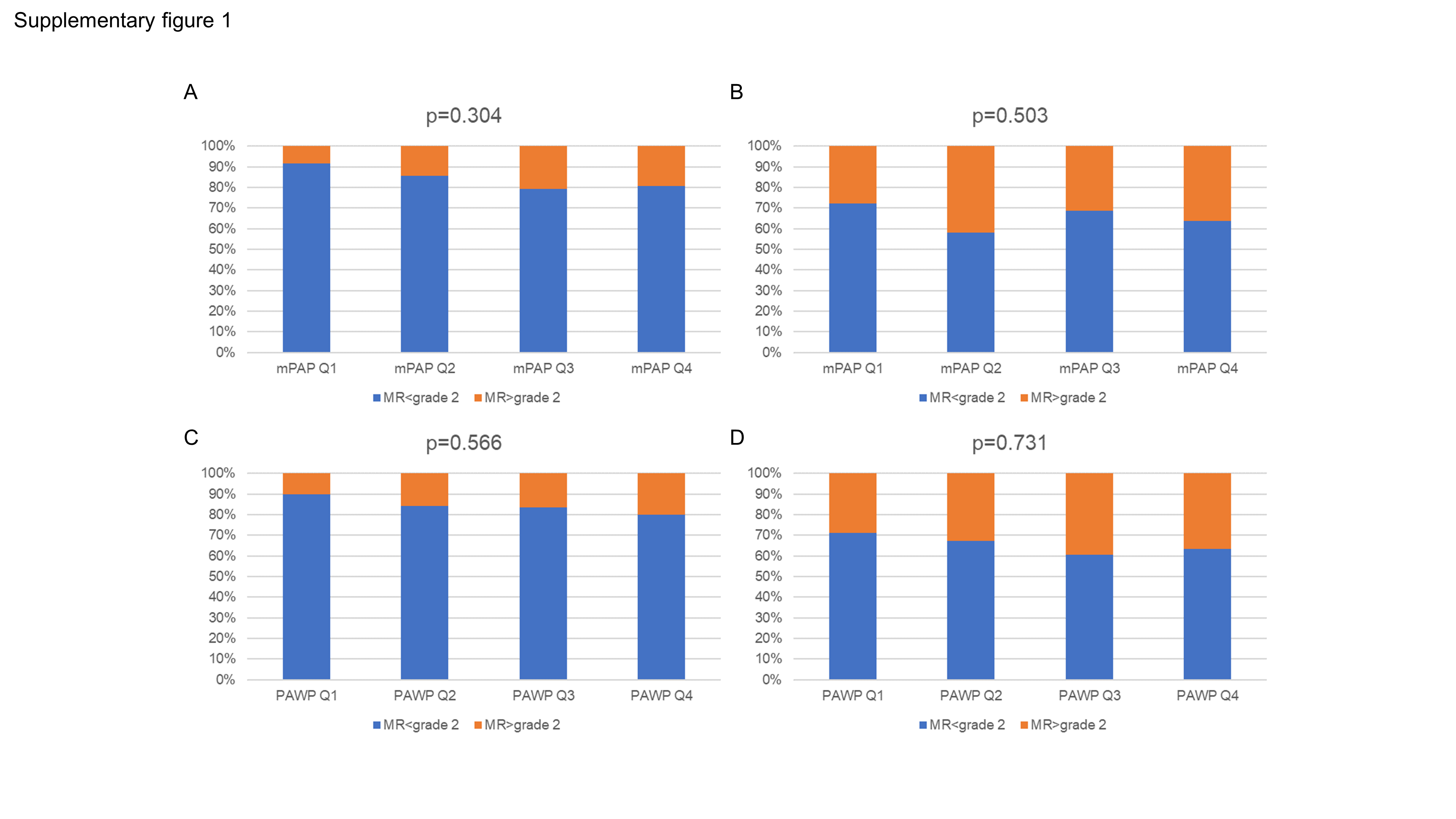

Supplement: Supplementary file 1 — Supplementary file1 (TIF 1157 KB) [file 392_2024_2442_MOESM1_ESM.tif]

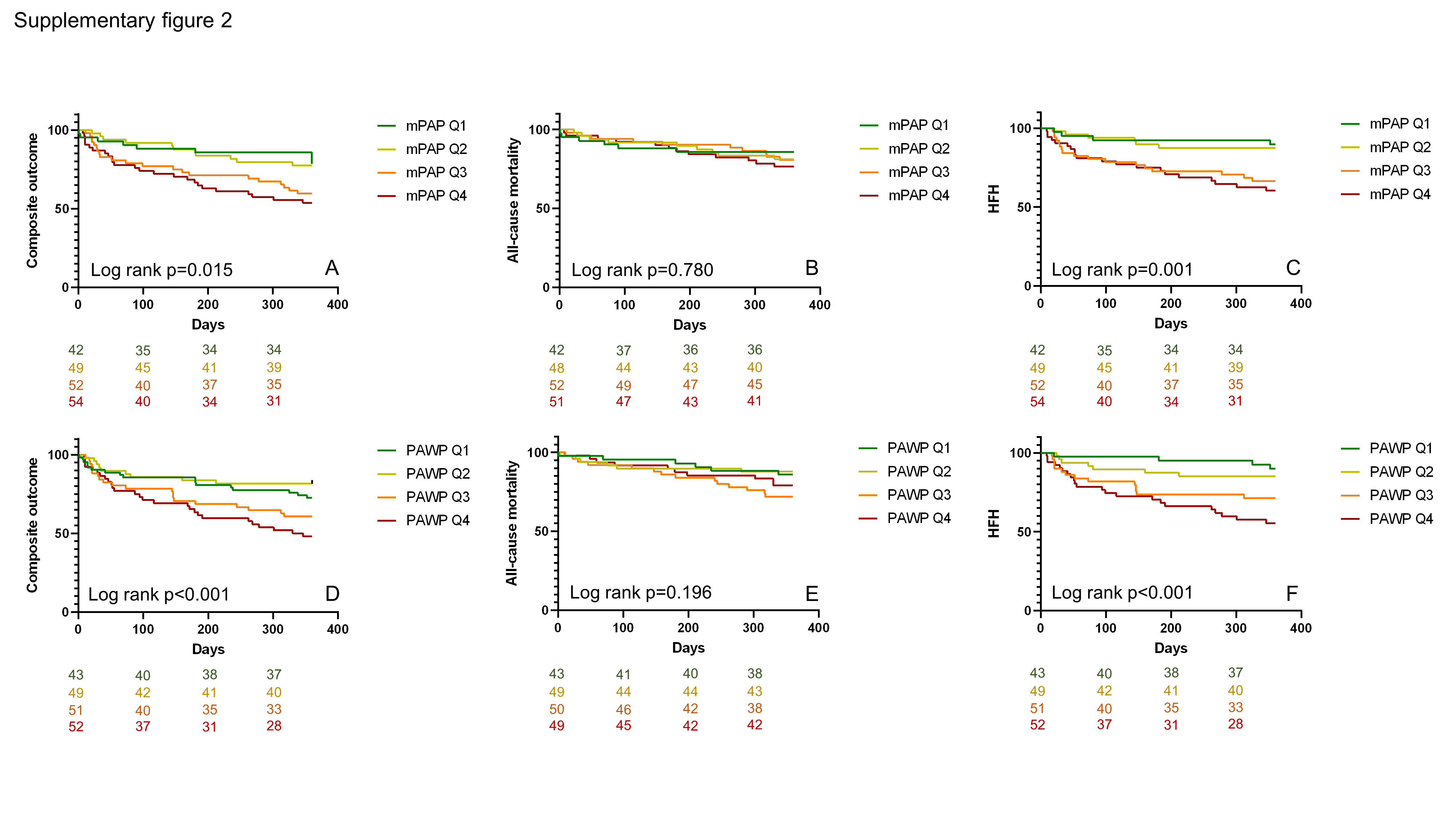

Supplement: Supplementary file 2 — Supplementary file2 (TIF 755 KB) [file 392_2024_2442_MOESM2_ESM.tif]
