# Supplementary material for: Impact of pulmonary hypertension on outcomes after TEER in patients suffering from mitral regurgitation
Source: Clin Res Cardiol. 2024 Apr 2;114(2):203–14. doi: 10.1007/s00392-024-02442-1 (PMC11839688; doi:10.1007/s00392-024-02442-1)
Supplement: Supplementary file 3 — Supplementary file3 (DOCX 15 KB) [file 392_2024_2442_MOESM3_ESM.docx]

**Impact of Pulmonary Hypertension on Outcomes after TEER in Patients suffering from Mitral Regurgitation**

Running title: Pulmonary Hypertension and Outcomes After M-TEER

Philippa Jaeger^1^, Ioannis Toskas^1^, Jessica-Kristin Henes^1^, Serhii Shcherbyna^1^, Frederic Schwarz^1^, Miriam Euper^1^, Peter Seizer^2^, Harald Langer^3^, Andreas E. May^4^, Tobias Geisler^1^, Meinrad Gawaz^1^, Jürgen Schreieck^1*^, Dominik Rath^1*^

^1^Dept. of Cardiology and Angiology, University Hospital Tübingen, Germany; ^2^Dept. of Cardiology and Angiology, Ostalb-Klinikum Aalen, Germany; ^3^Department of Medicine, Cardiology, Angiology, Hemostasis and Intensive Care Medicine, University Medical Center Mannheim, Germany; ^4^Medizinische Klinik I, Klinikum Memmingen, Germany

*These authors share last authorship

Address for correspondence:

Dominik Rath

Dept. of Cardiology and Angiology, University Hospital Tübingen, Otfried-Müller-Str. 10, 72076, Tübingen; Germany.

Phone: +49-7071-2982888, Fax: +49-7071-294474

Email: [dominik.rath@med.uni-tuebingen.de](mailto:dominik.rath@med.uni-tuebingen.de)

**Supplementary table 1:**

| Event | mPAP  1^st^ quartile | mPAP 2^nd^ quartile | mPAP  3^rd^ quartile | mPAP 4^th^ quartile | Log rank p |
| --- | --- | --- | --- | --- | --- |
| Composite outcome | 9/42/21.4 | 11/49/22.4 | 21/52/40.4 | 25/54/46.3 | **0.015** |
| ACM | 6/42/14.3 | 9/48/18.8 | 10/52/19.2 | 12/51/23.5 | 0.780 |
| Hospitalization due to heart failure | 4/42/9.5 | 6/49/12.2 | 17/52/32.7 | 20/54/37.0 | **0.001** |
|  | PAWP  1^st^ quartile | PAWP 2^nd^ quartile | PAWP 3^rd^ quartile | PAWP 4^th^ quartile |  |
| Composite outcome | 9/43/20.9 | 9/49/18.4 | 20/51/39.2 | 27/52/51.9 | **<0.001** |
| ACM | 6/43/14.0 | 6/49/12.2 | 14/50/28.0 | 10/49/20.4 | 0.196 |
| Hospitalization due to heart failure | 4/43/9.3 | 7/49/14.3 | 14/51/27.5 | 22/52/42.3 | **<0.001** |

*mPAP Q1* <24, *mPAP Q2* ≥24 <30, *mPAP Q3* ≥30 <37, *mPAP Q4* ≥ 37 mmHg

*PAWP Q1* <12, *PAWP Q2* ≥12 <18.5, *PAWP Q3* ≥18.5 <26, *PAWP Q4* ≥ 26 mmHg

**Supplementary table 2:**

| **Variable** | **HR (95% CI)** | **p (Composite outcome)** |
| --- | --- | --- |
| Hb | 0.83 (0.72-0.94) | **0.004** |
| CRT | 1.87 (1.01-3.45) | **0.045** |
| PAWP quartiles | 1.42 (1.11-1.81) | **0.005** |
| **Variable** | **HR (95% CI)** | **p**  **(HFH)** |
| PAWP quartiles | 1.76 (1.31-2.37) | **<0.001** |

Variables included into the model: Age, CMP, CRT, Hb, creatinine, MR, AS, TR, LVEF, mPAP, PAPsys, PAPdia, PAWP, a-wave and v-wave

***Classification of PH in patients with PVR available***

In patients with PVR available, we found that 188 patients suffered from PH (86.2%). Out of these patients, 52 (27.7%) suffered from pre-capillary PH, 50 (26.6%) from IpcPH and 86 (45.7%) from CpcPH. Within these subgroups, 1 patient without PH (3.7%), 8 patients with pre-capillary PH (18.1%), 12 patients with IpcPH (26.1%) and 26 patients with CpcPH (32.9%) were hospitalized due to heart failure.
